# Sleep duration and incident frailty: The Rural Frailty Study

**DOI:** 10.1186/s12877-021-02272-0

**Published:** 2021-06-16

**Authors:** Karla Moreno-Tamayo, Betty Manrique-Espinoza, Evangelina Morales-Carmona, Aarón Salinas-Rodríguez

**Affiliations:** 1 Unidad de Investigación Epidemiológica y en Servicios de Salud, Área Envejecimiento, Instituto Mexicano del Seguro Social, Cuidad de México, Mexico; 2 Center for Evaluation Research and Surveys, National Institute of Public Health, Cuernavaca, Morelos Mexico

**Keywords:** Sleep duration, Sleep complaints, Incident frailty

## Abstract

**Background:**

The association between sleep duration and frailty remains unconclusive since most of the studies have been cross-sectional. Therefore, this study aimed to analyze the association between sleep duration, sleep complaints, and incident frailty.

**Methods:**

A community-based cohort study from rural areas in Mexico with 309 older adults aged 70 and over. Data from waves two and three of the Rural Frailty Study were used. We operationalized the Fried frailty phenotype to describe prevalent and incident frailty at two consecutive waves. Sleep duration was classified as: ≤ 5 h, 6 h, 7–8 h, and ≥ 9 h; and the self-reported sleep complaints as a dichotomous variable. Analyses were performed using Poison regression models.

**Results:**

The average age was 76.2 years and 55.3% were women; the incidence of frailty was 30.4%; 13.3% slept ≤5 h, and 38.5% ≥ 9 h. Compared with the group that slept 7–8 h, the risk of frailty at 4.4 years of follow-up was significantly higher among those who slept ≤5 h (adjusted RR 1.80, 95% CI: 1.04–3.11) and among those who slept ≥9 h (adjusted RR 1.69, 95% CI: 1.10–2.58). Sleep complaints were not associated with incident frailty (adjusted RR 1.41, 95% CI: 0.94–2.12).

**Conclusions:**

Our results show that short and long sleep duration are associated with the incidence of frailty. Studies that objectively evaluate sleep duration are needed to clarify whether meeting the recommended hours of sleep decreases frailty incidence.

## Background

While it is true that aging is associated with changes in sleep, such as decreased total sleep time, lack of deep sleep, and waking up more at night, such changes are not a normal part of the aging process [1]. Optimal sleep, even in older age, is of key importance for overall health and wellbeing. In fact, evidence shows that older adults (OA) who experience sleeping problems can have a low quality of life, cognitive decline, depression, difficulty doing basic daily activities, as well as an increased risk of falls, cardiovascular disease, and even death [1]. Frailty increases susceptibility to adverse effects like disabilities and death [2], and tends to appear in later ages. Results from a meta-analysis indicate that after an average follow-up time of 3 years, 13.6% of OA not considered frail at baseline become frail [3].

Having low quality sleep and sleeping few hours, or too many, has serious physiological effects on the brain and profound biological stress on the cells, organs, and the body’s main regulatory systems. Therefore, it is plausible that certain sleep characteristics, like hours of sleep or sleeping disorders, could be related to incident frailty [4]. In fact, recent studies have linked sleep to frailty, including sleep complaints [5], sleeping less than the number of recommended hours [6–8] and/or sleeping too much [7–12], insomnia [6], or low quality of sleep [9, 12, 13]. Nonetheless, most studies are cross sectional and until now, the results have not been conclusive. The purpose of this study was to analyze the relationship between sleep duration, the presence of sleep complaints, and the risk of frailty in community dwelling older adults.

## Methods

### Study design and participants

Data from the second and third wave of the Rural Frailty Study that were collected in 2013 and 2018 were used. The Rural Frailty Study is a longitudinal study that began in 2009 and has three waves (2009, 2013, 2018). The methodological details have been described elsewhere [5, 14]. Briefly, it is a prospective cohort study whose principal aim is to determine the prevalence and incidence of frailty in older adults who reside in rural areas in Mexico. The sample size at baseline (2009) was 600 OA [14] and in each round, information was collected through interviews conducted in their homes. In each follow up measurement, a small refreshment sample was included to compensate for losses from mortality and follow up [5, 14]. The study was approved by the Research and Ethics Committee at the National Institute of Public Health. The OA signed the informed consent forms prior to data collection.

### Analytical sample

Of the 591 OA with complete information from round 2 (2013), 282 were excluded (47.7%) for being frail (*n* = 63, 10.7%), for having died during 2013–2018 (*n* = 77, 13%), or due to loss at follow up or having incomplete information for a variable (*n* = 142, 24%). For this study, we used a sample of 309 OA who, in 2013 (wave 2), were not considered frail. No statistically significant differences were observed between the OA included in our sample and those who were excluded for having incomplete information or for loss at follow up.

### Exposure: sleep duration and sleep complaints

Sleep duration was measured with the question “How many hours, on average, do you sleep in a day?” The responses were classified into four groups: 1) ≤ 5 h, 2) 6 h, 3) 7–8 h, and 4) ≥ 9 h, according to recommendations from the National Sleep Foundation [15]. Sleep complaints were assessed with the question “Have you had any trouble sleeping recently? and classified as a dichotomous variable (1 = response “yes”; 0 = response “no”). Sleep duration and sleep complaints were measured in 2013.

### Outcome: incidence of frailty

Incidence of frailty, defined as the new cases of frailty in round three of the study (2018), was measured following the frailty phenotype proposed by Fried et al. [2]. It evaluates the presence of the following criteria: Low strength, slow walking, exhaustion, low physical activity, and unintentional weight loss. The criteria to define frailty in the Rural Frailty Study have been described previously [14]. In summary, grip strength was measured with a dynamometer and walking speed was measured using a 4-m path. Exhaustion was evaluated with the questions *“Do you feel full of energy?” and “Do you have enough energy for your everyday life?”* Physical activity was determined with the *International Physical Activity Questionnaire*. Weight loss was measured as self-reported unintentional weight loss of ≥5 kg. The participants were classified as robust (0 criteria), pre-frail (1–2 criteria), or frail (3–5 criteria). For the analyses, frailty is considered a dichotomous variable (robust/pre-frail versus frail).

### Covariates

Sociodemographic characteristics like age (years), sex (female = 1), ethnicity (yes = 1), literacy (yes = 1), paid work (yes = 1), and marital status (married/cohabitating = 1). The presence of subjective memory complaints was determined with the question “Have you had any difficulty with your memory?” (yes = 1). Limitations in Activities of Daily Living (ADLs) was considered when the participant reported having difficulty doing at least one of the following: Showering, getting dressed, using the bathroom, moving around, continence, and eating [16]. The presence of limitations in Instrumental Activities of Daily Living (IADLs) was defined as having difficulty carrying out at least one of the following tasks: Using the phone, shopping, managing medications and money, using public and private transportation, and, in the case of women, preparing food and other domestic chores [17]. Function of the lower extremities was assessed with the *Short Physical Performance Battery (SPPB)*, which consists of three tests: Speed when walking, standing balance, and sit-to-stand performance. Scores for the SPPB range from 0 to 12; poor performance was considered < 8 [18].

The presence of chronic diseases was determined by asking the OA if they had a previous medical diagnosis of the following diseases (yes/no): Hypertension, diabetes, coronary heart disease, hypercholesterolemia, stroke, arthritis, and osteoporosis. The number of medications used by the participants was recorded for these conditions; the variable took on values from 0 to 7.

### Statistical analysis

The participant characteristics were described using means and proportions and chi-square or fisher exact tests were carried out to analyze the characteristics and incident frailty status. Chi-square or fisher exact test was also used to compare baseline health variables according to sleep duration. Various Poisson regression models were adjusted to estimate the association between sleep duration, sleep complaints, and incident frailty. First, an unadjusted model for each sleep variable (duration and complaints); second, a model controlling for sociodemographic variables (age, sex, education level, marital status, work, ethnicity); and third, a model that also controlled for health variables (subjective memory loss, limitations in ADLs and IADLs, physical performance, presence of chronic diseases, and number of medications). Differences were considered statistically significant if *P* < 0.05 and marginally significant if 0.05 *< P ≤* 0.10.

## Results

The average age of the OA in the second round was 76.2 ± 3.2 years and 55.3% were women. Accumulated incidence of frailty in the period 2013–2018 was 30.4% (*n* = 94). Sociodemographic and health characteristics of the OA by frailty status in 2018 are shown in Table 1. At baseline, a sleep duration of 7–8 h was reported by 35.6% of participants. The proportion of OA categorized with 5 h or less was 13.3 and 38.5% reported a sleep duration of 9 h or more. Characteristics of the OA by sleep duration are shown in Table 2. Diabetes seemed to be associated with sleep duration (*P* = 0.019); OA with long sleep or shorter duration were more prone to self-report diabetes. Sleep duration was not associated with other health conditions or demographic characteristics.

**Table 1:** Baseline characteristics of study participants according to incidence of frailty

Variables	Frailty at follow-up				***P*** Value
	Robust/Pre-frail		Frail		
	215	(69.6%)	94	(30.4%)	
	n	%	n	%	
Sleep duration (%)					
5 h or less	23	10.7	18	19.1	
6 h	30	13.9	9	9.6	
7–8 h	87	40.5	23	24.5	
9 h or more	75	34.9	44	46.8	0.009
Sleep complaints (%)	35	16.3	23	24.5	0.090
Women (%)	110	51.2	61	64.9	0.026
Ethnicity background (%)	75	34.9	43	45.7	0.071
Literacy (%)	92	42.8	30	31.9	0.072
Paid job (%)	79	36.7	19	20.2	0.004
With partner (%)	110	51.2	38	40.4	0.082
Subjective memory complaint (%)	54	25.1	40	42.6	0.002
Limitations in ADL (%)	23	10.7	26	27.7	*< 0.001*
Limitations in IADL (%)	39	18.1	31	33.0	0.004
SPPB, < 8 (%)	46	21.4	41	43.6	*< 0.001*
Hypertension (%)	72	33.5	41	43.6	0.089
Diabetes (%)	15	7.0	12	12.8	0.097
Hypercholesterolemia (%)	27	12.6	16	17.0	0.297
Coronary heart disease (%)	10	4.7	7	7.5	0.321
Stroke (%)	3	1.4	3	3.2	0.373
Arthritis (%)	22	10.2	10	10.6	0.914
Osteoporosis (%)	16	7.4	11	11.7	0.222
Mean age (years)	75.8	±3.04	77.01	± 3.48	0.002
Mean number of medications	0.44	±0.74	0.71	± 0.93	0.013

*ADL* Activities of Daily Living, *IADL* Instrumental Activities of Daily Living, *SPPB* Short Physical Performance Battery

**Table 2:** Baseline health variables of study participants according to baseline sleep duration

	Sleep duration								***P*** Value
	5 h or less		6 h		7–8 h		9 h or more		
	41	(13.3%)	39	(12.6%)	110	(35.6%)	119	(38.5%)	
	n	%	n	%	n	%	n	%	
Subjective memory complaint (%)	16	36.6	13	33.3	29	26.4	37	31.1	0.621
Limitations in ADL (%)	11	26.8	7	18.0	13	11.8	18	15.1	0.156
Limitations in IADL (%)	10	24.4	6	15.4	23	20.9	31	26.0	0.528
SPPB, < 8 (%)	8	19.5	10	25.6	29	26.4	40	33.6	0.313
Hypertension (%)	15	36.6	20	51.3	40	36.4	38	31.9	0.192
Diabetes (%)	4	9.8	4	10.3	3	2.7	16	13.5	0.019
Hypercholesterolemia (%)	5	12.2	7	18.0	16	14.6	15	12.6	0.840
Coronary heart disease (%)	3	7.32	3	7.7	5	4.6	6	5.0	0.740
Stroke (%)	0	0	0	0	2	1.8	4	3.4	0.629
Arthritis (%)	6	14.6	9	23.1	11	10.0	6	5.0	0.010
Osteoporosis (%)	6	14.6	4	10.3	7	6.4	10	8.4	0.402

*ADL* Activities of Daily Living, *IADL* Instrumental Activities of Daily Living, *SPPB* Short Physical Performance Battery

The group of OA who became frail, in a follow up period of 4.4 years, tended to sleep more or less than the recommended hours than OA who were pre-frail or robust (*P* < 0.01). Furthermore, the presence of sleep complaints was marginally greater in OA with incident frailty (*P* = 0.09). Differences in sociodemographic variables by incident frailty were observed. The percentage of women was higher in the group of frail OA than in the pre-fail and robust group (*P* < 0.05). The OA with frailty at follow up were older (*P* < 0.01) and less likely to have paid work (*P* < 0.01). In addition, the OA who developed frailty had more health problems than those who were pre-frail or robust, reporting more memory complaints (*P* < 0.01), limitations in ADL (*P* < 0.01) and IADL (*P <* 0.01*)*, poorer physical functioning (*P* < 0.01), and a marginally higher frequency of hypertension (*P =* 0.09) (Table 1).

The unadjusted Poisson regression model showed a strong relationship between sleep duration and increased risk of being frail at follow up (Model 1, Table 3), which persisted when sociodemographic covariates were controlled for (Model 2, Table 3) and in the completely adjusted model (Model 3, Table 3). When compared with OA who reported sleeping 7–8 h, the relative risk (RR) of being frail at follow up was 1.8 among those who reported sleeping 5 h or less (95% CI 1.04–3.11) and 1.69 for those who habitually slept 9 h or more (95% CI 1.10–2.58).

**Table 3:** Relative risks (95% confidence interval) for the association between sleep duration and sleep complaints on the incidence of frailty

	Model 1		Model 2		Model 3	
	RR	(95% CI)	RR	(95% CI)	RR	(95% CI)
Sleep duration (%)						
5 h or less	2.10*	(1.27–3.47)	1.78†	(1.04–3.06)	1.80†	(1.04–3.11)
6 h	1.10	(0.56–2.18)	1.03	(0.53–1.99)	0.99	(0.51–1.92)
7–8 h (reference)	1.00		1.00		1.00	
9 h or more	1.77†	(1.15–2.73)	1.66†	(1.07–2.56)	1.69†	(1.10–2.58)
Sleep complaints (reference: no)	1.40‡	(0.96–2.04)	1.42‡	(0.94–2.15)	1.41	(0.94–2.12)

Model 1: Unadjusted Poisson regression

Model 2: As Model 1 and additionally adjusted for sex (men, women), age (years), ethnicity background (yes, no), literacy (yes, no), paid job (yes, no), With partner (yes, no)

Model 3: As Model 2 and additionally adjusted for: subjective memory complaint (yes, no), limitations in ADL (yes, no), limitations in IADL (yes, no), SPPB (score ≥ 8, < 8), number of medications and presence of hypertension, diabetes, hypercholesterolemia, coronary heart disease, stroke, arthritis, and osteoporosis

* *P* < 0.01, † *P* < 0.05, ‡ *P* < 0.1

Sleep complaints were marginally associated with incidence of frailty in the unadjusted model (RR 1.40, 95% CI 0.96–2.04, *P* = 0.08). When controlling for age, sex, ethnicity, education level, work, and marital status, the relative risk was 1.42 (95% 0.94–2.15, *P* = 0.09). Lastly, in the model adjusted for sociodemographic and health variables, sleep complaints were not associated with incidence of frailty (RR 1.41, 95% CI 0.94–2.12, *P* = 0.10).

## Discussion

This study shows that sleep duration is associated with the incidence of frailty in OA (70 years and older) who live in rural communities in Mexico. In particular, it found that OA who sleep little (5 h or less) or too much (9 h or more) have an increased risk of incident frailty compared to those who sleep 7–8 h a day.

Consistent with our results, one study demonstrated that sleeping 6 h or less, or sleeping 9 h or more, is associated with a greater possibility of becoming frail [7]. Other investigations have reported that only short sleep duration (fewer than 6 h) [6, 8] or only long duration, increase the risk of frailty [8–12]. Nonetheless, these studies are based on cross sectional analyses. A longitudinal study with 3.4 years of follow up documented the negative effects of low quality sleep and nocturnal hypoxemia on frailty and mortality, but other indicators like sleep duration and latency did not have any effect on frailty or mortality [19]. To our best knowledge, this is the first prospective study that reports that both sleeping too little or too much are related to incidence of frailty at 4.4 years of follow up.

Although a cross sectional association was previously reported between sleep complaints and frailty in the same cohort of subjects [5], this relationship was not confirmed in the longitudinal analysis. It is likely that the longitudinal effect of sleep complaints is mitigated through other mechanisms, specifically due to the presence of unfavorable health conditions. In our study, we observed that by adjusting the variables related to health, the effect of sleep complaints on incidence of frailty was not maintained. Future research should use more objective and exhaustive measures to study sleep disorders and their association with incidence of frailty [20].

The mechanisms that underlie the association between short sleep duration and frailty involve disruptions in neuroendocrine regulation. Reductions in testosterone levels have been observed in individuals who report sleeping few hours, as well as chronic inflammation, greater oxidative stress levels, and growth hormone secretion imbalances, among others [4, 21]. Although the mechanisms behind long sleep duration are still not well understood, it is possible that its relationship with frailty can be explained as follows: Having biologically longer nights means longer periods with elevated melatonin and cortisol levels, as well as a lower body temperature [22], which can affect the immune system [23] and increase the likelihood of becoming frail. Morgan et al. (2018) state that OA who sleep a lot have low daily physical activity levels, reduced muscle strength, and slow walking speed [24].

In this work we studied frailty according to Fried’s phenotype model; however, other models have been developed; the deficit accumulation model is a widely recognized, it views frailty in terms of number of deficits that a person presents in various areas ranging from the physical (symptoms and diseases), as well as psychological and social domains. While Fried’s phenotype model predominates in the study of the link between sleep and frailty [20]; there are few studies that had employed the deficit approach. Balomenos et al. [12] found a cross-sectional relationship between sleep quality and frailty (frailty with three different measures: The Frailty Index (FI); the Tilburg Frailty Indicator (TFI), and the Groningen Frailty Indicator (GFI)) [12]. Del Brutto et al. [13] also documented an association between poor sleep quality and frailty using Edmonton Frail Scale [13]. Sleep duration showed a less clear association across frailty measures [12]. In the men sub-sample, long sleepers were more prone to be frail when it was evaluated by FI; sleep duration and GFI were only marginally associated [12, 25]. Thus, future research should explore sleep disorders in the context of different frailty approaches.

There is a notion that frailty prevention is possible. Majority of research has tested frailty interventions focusing on nutritional supplements, training programs and a combination of both [26]. There has been limited studies testing the effect of sleep interventions to prevent frailty. Our results highlight a potential for designing strategies to reduce frailty incidence by promoting healthy sleep hours, thus, adopting interventions focusing on sleep might potentially avoid progressing to frailty.

This study has various strengths. As far as we know, this is one of the first longitudinal studies with OA participants from Latin America. Frailty was evaluated according to international recommendations of Fried et al. and the study’s longitudinal design allows the relationship between sleep duration and incidence of frailty to be analyzed prospectively. However, the study also has some limitations. Although evaluating sleep duration with self-reports is a common practice in epidemiological community based studies, in the future more objective measures can be used like actigraphy. One important limitations of our study are that measures of sleep quality or sleep disorders were not evaluated, such as: naps during the day, fragmented sleep, the presence of excessive drowsiness, sleep apnea, among other alterations, whose inclusion may have strengthened our findings. Sleeping long duration, for example, could be associated with daytime sleep or with the presence of fragmented sleep. In particular, sleep fragmentation has been associated with decreased energy and increased function limitations [27]. At the end, healthy sleep is multidimensional, beyond the duration of sleep, as a recent study showed that regular snoring was associated with a lower chance of achieving healthy aging [28]. Heavy snoring may also lead to repeated arousals and fragmented sleep that disrupt sleep quality.

## Conclusions

This research adds to the scarce number of studies that have analyzed the relationship between sleep and frailty. Our results show that both short sleep duration and long duration are associated with incidence of frailty. More longitudinal studies are needed to clarify the role of sleep characteristics in frailty in older age.

## Data Availability

The dataset used and/or analyzed during the current study are available from the corresponding author on reasonable request.

## Abbreviations

OA: Older adults
ADLs: Activities of Daily Living
ADLs: Instrumental Activities of Daily Living

## References

[1] Miner B, Kryger MH (2017). Sleep in the aging population. Sleep Med Clin.

[2] Fried LP, Tangen CM, Walston J, Newman AB, Hirsch C, Gottdiener J, Seeman T, Tracy R, Kop WJ, Burke G, McBurnie MA (2001). Frailty in older adults: evidence for a phenotype. J Gerontol A Biol Sci Med Sci.

[3] Ofori-Asenso R, Chin KL, Mazidi M, Zomer E, Ilomaki J, Zullo AR, Gasevic D, Ademi Z, Korhonen MJ, LoGiudice D, Bell JS, Liew D (2019). Global incidence of frailty and Prefrailty among community-dwelling older adults: a systematic review and meta-analysis. JAMA Netw Open.

[4] Piovezan R, Poyares D, Tufik S (2013). Frailty and sleep disturbances in the elderly: possible connections and clinical implications. Sleep Sci.

[5] Moreno-Tamayo K, Manrique-Espinoza B, Rosas-Carrasco O, Pérez-Moreno A, Salinas-Rodríguez A (2017). Sleep complaints are associated with frailty in Mexican older adults in a rural setting. Geriatr Gerontol Int.

[6] Moreno-Tamayo K, Manrique-Espinoza B, Ortiz-Barrios LB, Cárdenas-Bahena Á, Ramírez-García E, Sánchez-García S (2020). Insomnia, low sleep quality, and sleeping little are associated with frailty in Mexican women. Maturitas.

[7] Nakakubo S, Makizako H, Doi T, Tsutsumimoto K, Hotta R, Lee S, Lee S, Bae S, Makino K, Suzuki T, Shimada H (2018). Long and short sleep duration and physical frailty in community-dwelling older adults. J Nutr Health Aging.

[8] Zhang Q, Guo H, Gu H, Zhao X (2018). Gender-associated factors for frailty and their impact on hospitalization and mortality among community- dwelling older adults: a cross-sectional population-based study. PeerJ.

[9] Sun XH, Ma T, Yao S, Chen ZK, Xu WD, Jiang XY, Wang XF (2020). Associations of sleep quality and sleep duration with frailty and pre-frailty in an elderly population Rugao longevity and ageing study. BMC Geriatr.

[10] Baniak LM, Yang K, Choi J, Chasens ER (2018). Long sleep duration is associated with increased frailty risk in older community-dwelling adults. J Aging Health.

[11] Kang I, Kim S, Kim BS, Yoo J, Kim M, Won CW (2019). Sleep latency in men and sleep duration in women can be frailty markers in community-dwelling older adults: the Korean frailty and aging cohort study (KFACS). J Nutr Health Aging.

[12] Balomenos V, Ntanasi E, Anastasiou CA, Charisis S, Velonakis G, Karavasilis E, Tsapanou A, Yannakoulia M, Kosmidis MH, Dardiotis E, Hadjigeorgiou G, Sakka P, Scarmeas N (2020). Association between sleep disturbances and frailty: evidence from a population-based study. J Am Med Dir Assoc.

[13] Del Brutto OH, Mera RM, Sedler MJ, Zambrano M, Nieves JL, Cagino K, Fanning KD, Milla-Martinez MF, Castillo PR (2016). The effect of age in the association between frailty and poor sleep quality: a population-based study in community-dwellers (the Atahualpa project). J Am Med Dir Assoc.

[14] Manrique-Espinoza B, Salinas-Rodríguez A, Salgado de Snyder N, Moreno-Tamayo K, Gutiérrez-Robledo LM, Avila-Funes JA (2016). Frailty and social vulnerability in Mexican deprived and rural settings. J Aging Health.

[15] Hirshkowitz M, Whiton K, Albert SM, Alessi C, Bruni O, DonCarlos L, Hazen N, Herman J, Adams Hillard PJ, Katz ES, Kheirandish-Gozal L, Neubauer DN, O’Donnell AE, Ohayon M, Peever J, Rawding R, Sachdeva RC, Setters B, Vitiello MV, Ware JC (2015). National Sleep Foundation’s updated sleep duration recommendations: final report. Sleep Health.

[16] Katz S, Branch LG, Branson MH, Papsidero JA, Beck JC, Greer DS (1983). Active life expectancy. N Engl J Med.

[17] Lawton MP, Brody EM (1969). Assessment of older people: self-maintaining and instrumental activities of daily living. Gerontologist.

[18] Guralnik JM, Ferrucci L, Simonsick EM, Salive ME, Wallace RB (1995). Lower-extremity function in persons over the age of 70 years as a predictor of subsequent disability. N Engl J Med.

[19] Ensrud KE, Blackwell T, Ancoli-Israel S (2012). Sleep disturbances and risk of frailty and mortality in older men. Sleep Med.

[20] Wai JLT, Yu DSF (2020). The relationship between sleep–wake disturbances and frailty among older adults: a systematic review. J Adv Nurs.

[21] Hall MH, Smagula SF, Boudreau RM, Ayonayon HN, Goldman SE, Harris TB, Naydeck BL, Rubin SM, Samuelsson L, Satterfield S, Stone KL, Visser M, Newman AB (2015). Association between sleep duration and mortality is mediated by markers of inflammation and health in older adults: the health. Aging and Body Composition Study Sleep.

[22] Aeschbach D, Sher L, Postolache TT, Matthews JR, Jackson MA, Wehr TA (2003). A longer biological night in long sleepers than in short sleepers. J Clin Endocrinol Metab.

[23] Patel SR, Malhotra A, Gottlieb DJ, White DP, Hu FB (2006). Correlates of long sleep duration. Sleep.

[24] Morgan K, Hartescu I (2019). Sleep duration and all-cause mortality: links to physical activity and prefrailty in a 27-year follow up of older adults in the UK. Sleep Med.

[25] Lachmann R, Stelmach-Mardas M, Bergmann MM, Bernigau W, Weber D, Pischon T, Boeing H (2019). The accumulation of deficits approach to describe frailty. PLoS One.

[26] Apóstolo J, Cooke R, Bobrowicz-Campos E, Santana S, Marcucci M, Cano A, Vollenbroek-Hutten M, Germini F, D'Avanzo B, Gwyther H (2018). Effectiveness of interventions to prevent pre-frailty and frailty progression in older adults: a systematic review. JBI Database System Rev Implement Rep.

[27] Guida JL, Alfini AJ, Gallicchio L, Spira AP, Caporaso NE, Green PA (2021). Association of objectively measured sleep with frailty and 5-year mortality in community-dwelling older adults. SLEEPJ.

[28] Shi H, Huang T, Ma Y, Heather Eliassen A, Sun Q, Wang M (2021). Sleep duration and snoring at midlife in relation to healthy aging in women 70 years of age or older. Nat Sci Sleep.

